# Mr. Ring, in Answer to Mr. Southams

**Published:** 1806-12

**Authors:** John Ring

**Affiliations:** New Street, Hanover Square


### *To the Editors of the Medical and Physical Journal.*

GENTLEMEN,

YOUR Correspondent, Mr. Southams, affords a striking illustration of the well known adage, that it is easier to give advice than to take it. He condemns hasty assertions, intemperate language, and abuse; yet within the compass of one short letter, he is guilty of them all.

He gives an opinion, that matter was not in fault, yet acknowledges it was dry; and that the inoculator was not in fault, though he used it in that state, in the inoculation of a person very difficult to be infected. He asserts, that intemperate language too often occurs in my writings on this subject, although he has not proved it; and although he confesses no such language occurred on this occasion. This is, therefore, intemperate language and abuse; an exemplification of the *suaviter in modo,* which he so much recommends; and a convincing proof that he preaches what he does not practice. As far as we can judge from this specimen of his ability, he may be a good calumniator; but he is a bad partisan.

I knew Mr. Fermor, as Mr. Southams supposes; and as he was a sensible man, am surprized that he suffered Mr. Southams to trifle so much with the health of his domestics, as to inoculate one of them once, and the other twice, with dry matter, when they had already been so often inoculated in vain.

I also sent Mr. Fermor my publications, as Mr. Southams remarks; and it is well known to his surviving friends, that so far from entertaining the same opinion of them which Mr. Southams entertains; he honoured them with the most flattering marks of approbation. He was indeed so blind to their defects, and so partial to the style of their author, that he declared, no one wrote like him; but this was when Mr. Southams had not taken up his pen.

Mr. Southams may think proper to interpret this encomium of Mr. Fermor in an ironical sense; but his conduct in other respects evidently proved, that however mistaken, he was sincere. He cultivated the friendship of their author, in a manner which he will ever remember with gratitude; and frequently sent him the most polite and pressing invitations, which nothing but his professional engagements, particularly his labours in the cause of vaccination, could have induced him to decline.

I need

I need not inform Mr. Southams, that the name of Fermor is renowned in English literature; that every author must have been proud to pay his respects to the representative of the favourites of the Twickenham bard; and anxious to visit an abode, venerable as the haunt of the Muses; and doubly venerable, as the abode of THE FIRST PATRON OF VACCINATION.

I have already given some account of the meritorious labours of this distinguished BENEFACTOR OF THE HUMAN RACE, in my Treatise on that subject; and embrace the present opportunity of doing farther justice to his memory, and to his virtues. Having occasion to visit Cheltenham, on account of the health of a relation, he became acquainted with Dr. Jenner, soon after the promulgation of his invaluable discovery; and frequently conversed with him on that topic; but was for some time inclined to be rather sceptical.

At length, however, he was convinced of the truth of Dr. Jenner's doctrine; and, in consequence of this conviction, invited his nephew, the Rev. Mr. Jenner, to pass a few months with him at Tusmore; and to inoculate the people in the neighbourhood with the cow-pock. Accordingly several hundreds of them were vaccinated; and vaccinated gratuitously, contrary to the interest, and, as I am informed, *to the inclinations,* of some persons practising in that part of the country; with whose names Mr. Southams is probably better acquainted than I am.

A considerable number of those who had undergone vaccination, were afterwards submitted to the test of inoculation for the small-pox, under the inspection of Doctor Wall, Sir Christopher Pegge, Doctor Williams, and other gentlemen of the University of Oxford and its environs, distinguished for their professional abilities; who, from the most liberal and humane motives, encouraged Mr. Fermor in his undertaking; and devoted much of their time to this very interesting and important subject.

The result of these experiments is detailed in Mr. Fermor's pamphlet, entitled, "Reflections on the Cow-pox;" the first publication in favour of the practice, written by any author out of the pale of the medical profession; which he did me the honour to send me. I have also given a statement of the result of those experiments in my Treatise, p. 274; where it will readily be discerned, that the effect of this test was not quite so trivial as some people ignorantly suppose, or as others artfully pretend; on the contrary, there was generally a local affection, sometimes times pain in the head or axilla, in one instance a pustule on the forehead, and in another one on the arm. These two pustules were probably occasioned by the application of matter to the parts, by means of the nails of the patients; since it is well known, that a pustule in the arm, in a person who has previously had either the small-pox or the cow-pox, is attended with an unusual degree of itching; and commonly provokes the patient to scratch.

From the analysis of Mr. Fermor's pamphlet, which I long ago laid before the public in the Treatise beforementioned, and from his other publications, it appears, that he was a man of unbounded benevolence; of an enlightened and comprehensive mind; and anxious to promote the welfare and happiness of the whole human race. Such are the characters whom reason and philosophy delight to honour:

Quique sacerdotes casti, dum vita manebat,
Quique pii vates, et Phoebo digna locuti,
Inventas aut qui vitam excoluere per artes,
Quique sui memores alios fecere merendo.
Hæc tibi semper erunt, et quum sollemnia vota
Reddemus Nymphis, et quum lustrabimus agros.
Dum juga montis aper, fluvios dum piscis amabit.
Dumque thymo pascentur apes, dum rore cicadæ,
Semper honos nomenque tuum, laudesque manebunt.
Ut Baccho Cererique, tibi sic vota quotannis
Agricolæ facient; damnabis tu quoque votis.

---

I shall here take the liberty of inserting two letters, now in my possession, received from Mr. Fermor; which afford ample proof, that he could discover something in my writings, besides "*intemperate language*," or "*warmth and asperity*." These he left to be discovered by Mr. Southams; or by some discomfited opponent, in the disguise of a Reviewer.

"Sir, "*Tusmore, near Brackley, Dec.* 21, 1801

"I have lately received from you a Treatise on the cow-pox; for which I beg leave to return you my best acknowledgements. I can with truth aver, that I know not which to value most, the accurate account you have given of the rise and progress of this disorder; or the science you have displayed in so doing. There is only one fault which I can find in your publication; and that is, where you speak too favourably of the poor efforts which I have made to extend the practice.

"I am

"I am thoroughly convinced, that too much cannot be said in its commendation; and that we shall live to see" (alas! he did not live to see) "that hydra the small pox completely subdued, by the powerful arm of a Jenner; and the word to be found only in dictionaries.

"When I said in my publication, that I considered the cow-pox and the small-pox as synonymous terms, I meant to say, that whoever had undergone the one, was insusceptible of the other. How far the one is a modification of the other, I cannot pretend to say; but I am strongly inclined to think, as, I perceive by your publication, some others are disposed to do, that there is a great analogy between the two disorders. We have a right to conjecture that the cow-pox is the seed or germen of the small-pox, innocent at its origin, but becoming virulent by passing so frequently through "the gates and alleys of the human body," as Shakspeare beautifully expresses it; and in consequence becoming a partaker of the humours and disorders incident thereto. Thus it may ultimately degenerate into the worst sort of small-pox; of whose origin our great Sydenham candidly confesses himself ignorant. In that case, we must again have recourse to its uncontaminated source—the cow.

"In a tour which I made to Bath last summer, I had frequent opportunities of seeing Dr. Haygarth; who told me he was of opinion, that parliament ought to grant Dr. Jenner a remuneration; and that a petition for that purpose should be signed by every medical man in England.—What a just idea! and how admirably conceived! I most ardently hope, that honours will attend him as well as emoluments; and that our University of Oxford will confer on him a diploma. With what pleasure should I announce this news to him, in the following applicable lines,

> Aggredere, o magnos, aderit jam tempus, honores,

> Clara Deum Soboles! magnum Jovis Incrementum!

or a man chosen by God, to alleviate the miseries of mankind; and to repair the incalculable damages committed by an almost universal destruction of the human species, by an unparalleled and unprincipled war!

"I wish I could be so fortunate as to see you here; and have it in my power personally to assure you, how much I am, Sir,

Your obedient and humble servant,
Wm. Fermor.

"P.S.

"P.S. I have taken the liberty of enclosing for your acceptance, a copy of my publication, the binding of which may perhaps recommend it to a corner in your library."

The publication here alluded to was that on vaccination; I some time after received another from him, on one of the most important objects of agriculture. These, and his other laudable and scientific pursuits, justly entitle him to the encomium which I have paid; and prove that he was equally intent on promoting the necessaries and the comforts of life; the being and the well-being of man.

In his second letter, dated October 23, 1803, he has also greatly over-rated my poor services, and humble endeavours to serve the community. He says,

"Dear Sir,

"I have perused, with much satisfaction, the second volume of your work on vaccination. Were kings as anxious to preserve the lives of mankind, as you have shewn yourself to be, we should not see the world devastated, as it now is, by unnecessary wars, and absurd destructive expeditions. How fortunate is it, in the midst of such horrors, to be convinced, that one of the greatest enemies of mankind, the small-pox, is setting, never to rise again!

"What infinite pains you have taken, to lop off every head of the hydra as soon as it appeared! There is scarcely an alley in the metropolis, or its environs, which you have not penetrated and explored, courting contagion.

Quæ regio in terris vestri non plena laboris?

"I have lately been at Cheltenham; and seen that Benefactor of Mankind, Doctor Jenner. All the world must unite in rewarding a man, who has made, and will make, so many happy. Mens sana in corpore sano is the ultimatum of every thinking man's wish.

"I think it would be a very laudable scheme, to endeavour, by some means or other, to establish an Universal Subscription for him. It might be done by means of Consuls, in different parts of the globe; and be much promoted through the medium of our over-grown East India Company, the African Company, and others. These different societies might direct their agents, to make the proposal to the governments and individuals where they reside. The African heart must soften at such a proposal, and cry out, Non obtusa adeo gestamus pectora Poeni, as to refuse you; and conclude with Dido, Opibusque juvabo.

"Pray

"Pray tell me, whether you think these are the ravings of a man excluded from the world, and a hermit in the true sense of the word; or whether you are of opinion, that this plan might be reduced to practice. When you have sufficient leisure from your many useful avocations, I shall be happy to hear from you. I wish I could assure you personally, how much I am,

"Dear Sir,

"Very sincerely your's,

"Wm. Fermor.

"P.S. Every thing considered, do you not think we are more obliged to the horse, than to the cow? At least, Dr. Loy must be of this opinion. I like your motto much."

Not so Mr. Southams; he neither likes my motto nor my book: which, according to his statement, contains hasty assertions, and intemperate language. I should be sorry, however, if they contained so many unmerited aspersions, as Mr. Southams has crowded into a single page.

Had I consulted my own interest, I should certainly have witten in a more conciliatory strain; or at least, if I had occasionally ridiculed either or both parties, in order to recommend myself to the public, I should have taken care not to open too wide a breach between those who were raised to some degree of eminence in the profession, however undeservedly, and myself. They resemble certain noxious vermin, and venomous reptiles; which, if they are not powerful to do good, are powerful to do mischief. I have abundantly verified the old Terentian adage.

Obsequium amicos, veritas odium parit.

When I contemplated the conduct of those miscreants, who have so long been deluding the public, by asserting many things which they know to be false, and many others which they did not know to be true, in order to prejudice them against vaccination, it would have been criminal to suppress my feelings, and not give vent to those sentiments of indignation, which such nefarious proceedings were naturally calculated to inspire. No man, who took this decided part, could fail to incur some degree of odium among the enemies and the lukewarm friends of the practice; or to incense that party, whose treason against human nature he had exposed. I have seen, in the hands of Dr. Thornton, a letter from one of the opponents of vaccination; in which he warns those who write any thing against him, to be more cautious in future, *if*

*they*

*they regard their own personal safety.* This is the language of an assassin.

But there are assassins of a blacker dye; the assassins of reputation. These, either from private pique, or jealousy, retire to the lurking-hole of a Review, and there endeavour to gratify their malignity. I am ready to admit, that some publications of this kind are conducted on fair and honourable principles; but others are a disgrace to our age and country; and would be a disgrace to any age or country that gave birth or encouragement to such a farrago of slander and falsehood.

The author of one of them seems to fancy that he has a patent for abuse; and if any other man encroaches on his prerogative, and passes a censure, however justly merited, he takes care to let him know, that he has a right to a monopoly of this article.

It is true, some of the works on vaccination were published in haste, and in a crude state. It is true they are a sort of chaos; but their author, who was induced to accelerate their publication, from motives of humanity, was as sensible of this imperfection in those works as any mercenary scribbler can be; and has not only apologized for it in his prefatory remarks, but also, as far as possible, supplied the want of arrangement by a copious index. It is, however, some consolation to reflect, that this chaos has furnished materials for every subsequent work on vaccination: and that out of this chaos has arisen the beautiful order, in this new creation of medical science, which we now behold.

It is also true, that no critic, in the whole course of his censorial labours, could have "had occasion to contemplate a scene so disgusting and humiliating, as is presented by the greater part of this controversy; and "that no political or personal animosity, even among the lowest and most prostituted scribblers," ever gave rise to so much coarse and intemperate language, gross scurility, and low vulgar abuse, as has been exhibited by the opponents of vaccination, except in the purlieus of Grub-street, or the virulent and malignant page of an EDINBURGH REVIEW.

But whatever imperfections those works in favour of vaccination may labour under, it will be admitted as an ample apology by every candid critic, that they were written amidst an uncommon pressure of professional avocations, and a variety of disappointments from the different artists employed in the execution of the plates; which rendered it necessary, either to print the practical and and historical parts alternately, or to omit some of the most important information. Such as they are, they are submitted to the tribunal of the public; and will be handed down to a grateful posterity; while the malignant critic, who vainly endeavours to blast their reputation, " struts and frets his hour, and is forgotten."

It is with an ill grace a man accuses others of " personality and intemperance," whose personality and intemperance far exceed his critical acumen; who tries to blast the reputation of others, in order to raise his own; and is such a slave to national prejudice, that he can seldom discover merit in any one, who was not born on the other side of the Tweed. He seems to fancy, that in this part of the kingdom, learning is eclipsed, and genius extinct; that we have all been groping in the dark; and that we have nothing to direct us, in the paths of literature, or the walks of science, but the faint and glimmering coruscations of the *northern lights.*

To return to Mr. Southams, it is difficult to prove a negative and to refute a general charge; but I humbly presume, in addition to Mr. Fermor's testimonial, it will be sufficient to adduce that of a celebrated professor, the physician who attended his person, in opposition to that of Mr. Southams, who attended his household.

*Copy of a Letter from Dr.* WALL *to the Author, dated Oxford, March* 29, 1802.

" SIR,

" I ought long ago to have thanked you for your obliging present of the First Part of your Treatise on the Cow-pox; but a great variety of engagements prevented me.

" I have been extremely pleased with the perusal of your impartial history of this invaluable discovery; and your firm and manly defence of it against calumny and detraction. I hope the general voice of the nation, speaking in parliament, will give its sanction to the merits of our excellent friend Dr. Jenner, by remunerating in the best way it can, for it can never render an adequate compensation, for the eminent services which he has conferred upon the state.

" From the day when I first received intimation of Dr. Jenner's discovery, of the effect of inoculating the vaccine, to the present hour, I have never once varied in my opinion; though cases exciting scepticism in some gentlemen, have occurred in this neighbourhood. Many of them have have been explained; and the others might, I have no doubt, had it been possible to develope the whole progress of the several cases.

"Many years ago I learnt, in or by my connexions in Gloucestershire, that those who have had the casual cow-pox could never have the small-pox. The fact, however singular, lay treasured up in my mind; and when Dr. Jenner's discovery came to my knowledge, it met a mind prepared and ready to receive this extraordinary communication, which to most people appeared a dream, or an invention of fancy.

"Accept, Sir, my thanks for the exertions you have made in this great cause; and, though I have not the pleasure of your personal acquaintance, assure yourself that I feel every sentiment of regard for a gentleman, who has proved himself so eminently the promoter of medical science, directed to its best end, the preservation of the lives of millions."

Such testimonials as these induced me to believe, that my efforts to promote vaccination had not been altogether unsuccessful; and I should still be inclined to cherish that fond hope, did not some kind monitor now and then make his appearance in print, in order to dispel the delusion. This may serve as a warning to me in future. Rome was saved by the cackling of a goose.

This Journal bears witness, that if I have injured the cause of vaccination, and the cause of humanity, which I intended to serve, another physician of the first celebrity, besides Dr. Wall, has committed the same mistake, and betrayed the same want of judgment with him. In vol. 8, p. 546, Dr. Brandreth informs Dr. Marshall, that all the medical practitioners of Liverpool, who stand high in character, then recommended the cow-pock; and had adopted it, with full confidence of its being an effectual security against the small-pox.

He represents the practice as rapidly spreading in the neighbourhood, and likely to become universal. He maintains, that the efficacy of it was then indisputably established; that the safety with which it may be performed at any age, and under every state of health, must overcome every obstacle, and silence the cavils of ignorance, prejudice, and malevolence; that it must prevail, and cannot fail to be esteemed the most valuable blessing that ages and centuries have bestowed on the human race. But, he adds, "it is wholly unnecessary to repeat the advantages which must accrue from this discovery, since th they have already been so ably illustrated by Mr. Ring; whose Treatise on this subject is a master-piece; written with zeal, candour, and great knowledge of the subject.

"The mass of evidence which he has produced in its favour would convert an infidel; and I peculiarly admire the ingenuity and success with which he has detected the sources of unfavourable reports; and laid before his readers, in the compass of a moderate volume, all the knowledge, and an analytical review of all the publications on this interesting subject.—I can add nothing useful."

He concludes with expressing his most ardent wishes, that Dr. Jenner, who had so candidly and so readily communicated to the public his knowledge on this subject, might receive an ample and honourable remuneration.

If testimonies like these are not sufficient to shield my writings from the shafts of satire, and the breath of calumny, I know not what is. Perhaps, those gentlemen who cast such invidious reflections on them, may think proper to make a distinction between my Treatise and the answers which I have published to the late virulent attacks. As far, however, as I can trust to the testimonies of the most eminent members of the profession, those answers are a complete refutation of the charges brought forward by our opponents. From one very eminent physician I have received an opinion, that they abound with well-directed irony, and sound argument; and from another, that they contain the best piece of medical criticism which he has ever seen.

After all, if they possess but half the merit, which the partiality of these gentlemen ascribes to them, it may possibly be admitted as some atonement by the world at large; and accepted as a peace offspring by every critic, except one, who has been often defeated in the open field of literature, and, in order to gratify his resentment, opens a masked battery in a Review.

Let the gall'd jade wince.

The following testimonial alone is an ample remuneration for my labours. It may also help to quiet the apprehensions of those kind monitors, who feel a more tender concern for my reputation than for their own; and are fearful that I have "injured the cause which I meant to serve."

"Royal Jennerian Society for the Extermination of the Small-pox.

"At a Medical Council, October 6, 1803,

"The

"The Council considering, that after the promulgation of the discovery of vaccination, many obstacles had occurred to the extension of the practice, to the removal of which Mr. Ring contributed in a particular manner, by his assiduity and influence, his writings and his successful practice; by which he promoted and extended vaccination through the metropolis, as well as most parts of Europe; under this conviction, the Medical Council recommend to the Board of Directors, to confer on him some signal mark of approbation, for his laudable and distinguished services.

"At a Board of Directors, Dec. 1, 1803,

"Resolved, that the pamphlets of the Society, and the works of Dr. Jenner and Mr. Ring, together with vaccine matter, be sent to his Excellency Lieutenant General Nugent, Lieutenant Governor and Commander in Chief of the Island of Jamaica."

"At a Quarterly General Court, March 7, 1804,

"John Ring, Esq. was proposed, and unanimously elected, a Vice President of the Medical Council."

The Society also did me the honour to send my Treatise, the only work which I had then published on vaccination, together with that of Dr. Jenner, and such others as they approved of, to the Governor General of India, and the Governors of Madras and Bombay; in consequence of which, I have been regularly favoured with the many important publications on the same subject, in that remote quarter of the British dominions, and the most flattering testimonials from their authors. I have, therefore, nothing more to wish, with regard to medical honours: and if I cannot exclaim in triumph,

Urbem præclaram statui, mea mœnia vidi,

I may, at least, be permitted to indulge the pleasing reflection, that my humble exertions have been crowned with success; and that I have not written in vain.

---

It is now incumbent on me to answer another letter in the last Number of the Journal from Mr. Key, who thinks I have mistaken his meaning, while I think he has mistaken mine. Mr. Key authorized me to publish the documents or not, as I thought proper; and it was at first my intention to publish them at length; not from any conviction of my own that they were of much weight, but out of respect to Mr. Key, and lest it might be supposed that I

 had had any improper motive for suppressing them, or publishing them in a concise form. But when I saw, in addition to the accumulated evidence, which I have on so many occasions already brought forward, the strong and decisive testimony of Dr. Willan, in opposition to the inference which might be deduced from those cases, I thought them of still less importance; and was inclined to believe, that it was the less necessary to give them in detail.

The very flattering manner in which Mr. Key made this communication was such, that if he had been an indifferent person he must have won my regard; but being a man whom I have long been proud to call my friend, whom I have ever held in the highest esteem, and from whom I have received the most decisive marks of friendship, I could not possibly intend, by the mode in which I introduced his name, to cast the least imputation upon him. On the contrary, I should have been glad, as I now am, of an opportunity of paying him that tribute of respect to which his early, zealous, and able services, in the cause of vaccination, are so justly entitled.

In the second volume of my Treatise, published more than three years ago, p. 721, is the following attestation in favour of the practice, which I had some time before received from him:—" When I commenced vaccine inoculation, it was my intention to have published the result of my practice; but in 253 cases, under my direction, there was so little variety, and every circumstance was so favourable, that my adding my name to any testimonial in favour of the new practice, will be saying every thing that is requisite. Indeed, so much has been written, that unless something out of the usual way should occur, if every practitioner will annually register his testimony, it will be sufficient to impress upon the public the importance of the obligation due to Dr. Jenner. You have, therefore, my permission, to put my name to any treatise in favour of the disease."

In his note, accompanying the cases of supposed failure, Mr. Key stated, that he submitted them to me, " as the best friend of vaccination;" so much was he blinded by his partiality, and warped in his judgment. He did not then know, what some sagacious gentlemen have lately told the world, that it was a doubtful point, whether I had served the cause more by my unremitting exertions, than I had injured it by my intemperate zeal.

I never entertained the least doubt, that Mr. Key had collected collected the cases with care, nor that his veracity was to be implicitly relied on; but as the cases did not occur in his own practice, I thought it the more probable, that some mistake might have taken place, either with regard to the first or the second affection.

Mr. Key says, the cases appeared to him to contain occurrences deserving of consideration. This I allow, and under this impression, as he submitted them to me, I considered them. The more I considered them, the less important they appeared to me; and particularly after the appearance of Dr. Willan's testimony, in confirmation of my own. As far as they go, however, they tend to prove, that the effect of vaccination is only temporary. When, therefore, Mr. Key feels hurt at my representing this tendency, or purport, as of the intention of the communication, he appears to me to have mistaken my meaning.

Had I even asserted, that *his* intention, in communicating the cases, was "to bring forward evidence in addition to what has been already advanced by some authors, that vaccination is only a temporary protection against the small-pox;" I cannot see how it could reasonably be considered as any reflection on his integrity, or his judgment. On the contrary, it was natural to suppose, from the manner in which he communicated the cases, that he was influenced by conscientious motives, thinking them of some weight; or by a desire of satisfying the parents, and convincing them, that the practice is founded on just principles, and needs no concealment.

Fully persuaded that he was not induced to take this step by any but the most honourable motives, had he really been staggered in his faith, with regard to vaccination, it would not prove that he was not sincere in any opinion which he had previously given respecting the practice; nor would it have been an indispensable duty to relinquish it at once, till he had collected the general sense of the principal inoculators on that point. Consequently, those parents, to whom he has heretofore recommended it, when under a conviction of its affording a permanent security, could have no reason to complain.

I was far from suspecting, that Mr. Key wished to depreciate vaccination; or that he entertained an idea of publishing them, but as a painful duty.—I was far from suspecting, that he was one of those malignant spirits, who grieve at its prosperity, and strive to effect its downfal.—I have the less reason, however, to regret this misunderstanding, from whatever cause it has arisen, since it furnishes nishes the most incontestable proof, that Mr. Key still holds this beneficial process in the highest estimation; and that he is indignant, at being suspected to be an apostate from the practice.

I am, &c.

*New Street, Hanover Square,*
*November* 20, 1806.

JOHN RING.

